# Geoprocess of geospatial urban data in Tallinn, Estonia

**DOI:** 10.1016/j.dib.2023.109172

**Published:** 2023-04-22

**Authors:** Nasim Eslamirad, Francesco De Luca, Kimmo Sakari Lylykangas, Sadok Ben Yahia, Mahdi Rasoulinezhad

**Affiliations:** a FinEst Centre for Smart Cities, Tallinn University of Technology, Tallinn, Estonia; b Department of Civil Engineering and Architecture, Tallinn University of Technology, Tallinn, Estonia; c Department of Software Science, Tallinn University of Technology, Tallinn, Estonia

**Keywords:** Urban data acquisition, Urban analysis, Urban Heat Island (UHI), Ascending hierarchical grid system, Urban heterogeneity

## Abstract

The new digital era brings increasingly massive and complex interdisciplinary projects in various fields. At the same time, the availability of an accurate and reliable database plays a crucial role in achieving project goals. Meanwhile, urban projects and issues usually need to be analyzed to support the objectives of sustainable development of the built environment. Furthermore, the volume and variety of spatial data used to describe urban elements and phenomena have grown exponentially in recent decades. The scope of this dataset is to process spatial data to provide input data for the urban heat island (UHI) assessment project in Tallinn, Estonia. The dataset builds the generative, predictive, and explainable machine learning UHI model. The dataset presented here consists of multi-scale urban data. It provides essential baseline information for (i) urban planners, researchers, and practitioners to incorporate urban data in their research activities, (ii) architects and urban planners to improve the features of buildings and the city, considering urban data and the UHI effect, (iii) stakeholders, policymakers and administration in cities implementing built environment projects, and supporting urban sustainability goals. The dataset is available for download as supplementary material to this article.

## Graphical Abstract

**Figure:**
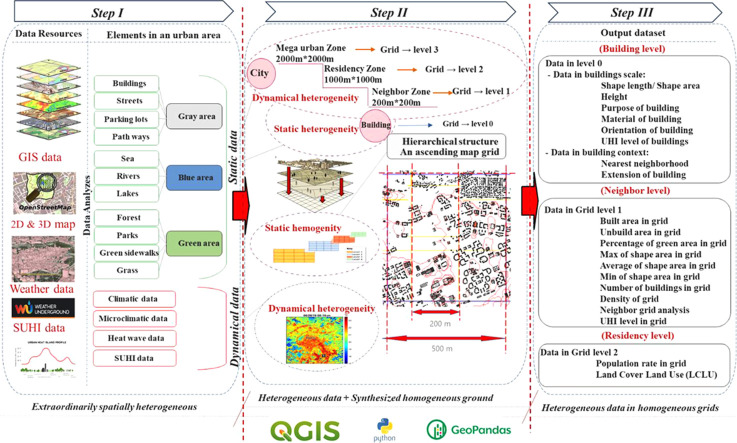

**Specifications Table**

**Table:** 

Subject	Architecture
Specific subject area	Architecture and urban planning, built environment, urban heat island (UHI) assessment
Type of data	Vector data Table
How the data were acquired	Data were acquired via geoprocessing, programming, and analysis. Applying an ascending hierarchical grid system is based on the theory of dynamic urban heterogeneity and considers data schema, features, and location. Data were processed using Python programming packages and the QGIS Tool for geoprocessing and analysis.
Data format	Raw and analyzed
Description of data collection	An extensive multidisciplinary presented dataset is collected with 34, 001 building samples (raws) and 30 features (columns) in Tallinn, including location, building characteristics, urban characteristics, UHI data, and climate data. The current work methodology proposes a framework to categorize data into homogeneous or heterogeneous, static or dynamic schemes, and then collect data considering the homogeneous grid system. Implementing the hierarchical grid system in the data collection process helps create a spatial index for each object and connects the objects to the grid system. Second, use the homogeneous ground to define urban indices mainly anchored in the heterogeneous data. The methodology uses the Python, Numpy, Pandas libraries, the Geopandas package, and QGIS Tool. The approach helps to capture urban data from GIS resources, taking into account the location, general characteristics, and other spatial properties of urban elements.
Data source location	• City: Tallinn, Country: Estonia • Latitude and longitude for collected data: 59.436962 and 24.753574 [1] • Domain size (km2): 159 km2 area [2]
Data accessibility	https://geoportaal.maaamet.ee/eng/Spatial-Data/Cadastral-Data-p310.html https://veeb.tallinnlv.ee/pilv/index.php/s/gdqlbwwbT7Ocqv3 Repository name: Mendeley Direct URL to data: https://data.mendeley.com/datasets/gwpbktrx9g/1 Direct URL to images: https://data.mendeley.com/datasets/xm92bw2f49/1 Direct URL to codes: https://github.com/maraso-TTU/Urban-Data

## Value of the Data

• The usefulness of the presented dataset lies in its ability to provide a comprehensive understanding of the urban environment by incorporating various essential features related to buildings, their surroundings, and meteorological and climatic data, with a specific focus on the UHI effect.
• The data can benefit a wide range of stakeholders, including architects, urban planners, policymakers, and local authorities, who can use the information to monitor urban data, set mitigation strategies, and improve the urban quality and quality of life of future development projects.
• Moreover, the data can be reused for further insights and development of experiments in various research fields, such as environmental studies, urban sustainability, and built environment studies, to understand better the UHI effect and its impact on the urban environment. The presented workflow and method can also be replicated in other comparative studies to collect and analyze relevant spatial data.
• Overall, not only the presented dataset has significant potential to contribute to the development of sustainable and liveable cities by providing detailed information on the urban environment, which can inform decision-making and policy development, but also the presented workflow and method allow researchers to extrapolate building and city data, including location-based features and metrological conditions with urban data and UHI intensify.

## 1 Objective

Geographic information systems (GIS) are now widely used, data-intensive disciplines for managing, displaying, and developing data queries on maps. One of the biggest challenges for urban planners remains the transformation of data into knowledge to facilitate planning efforts in addressing issues of complex urban systems, which requires advanced interdisciplinary analytical methods [3]. Therefore, there is a constant need for efficient methods, analytical tools, and rational design methods. GIS is characterized by various spatial and numerical interactions of geographical objects compared to other computational tools that can present data in tabular form [4].

In the data capturing method, some solutions, such as hierarchical principles, widely used in various disciplines to split complex problems into smaller ones, are applied to consider heterogeneous urban data in a homogeneous base. For the first time, the hierarchical system is defined as Hierarchical Spatial Reasoning (HSR) based on Car's spatial theory in 1997 [5]. Hierarchical principles allow the Part-Whole system to apply to all elements within the hierarchical system, forming a part of the whole [5]. Each level connects the lower and the higher levels [5]. Furthermore, spatial heterogeneity is often assumed to result from large-scale regional effects or administrative subdivisions that narrow the scope of a process [2].

## 2 Data Description

The initial available data are divided into three groups: Urban, Weather and climate, and UHI. The urban data are mainly downloaded from the Tallinn Land Authority Geospatial Data Portal (Maaamet), owned by the Estonian state, local governments, and legal persons governed by public laws [6]. Data formats are Spatial data (ESRI shapefile), Shapefile (.SHP), and Shape Index file (.SHX) and indicate data of buildings and land information, green areas, landscape, streets, blue bodies, and details of facilities and infrastructures in the city of Tallinn. For example, according to the Data Portal of Tallinn (Maaamet), the number of buildings located in Tallinn is 67, 113 [6].

The data relating to Surface Urban Heat Island (SUHI) is the output of the study that used Landsat-8 images suitable for the UHI effect studies and Land Surface Temperature (LST) data [7]. The authors used Landsat-8 to assess the heat wave's impact on Estonian cities and explore the extent and magnitude of the UHI effects.

Furthermore, the air temperature used to define the threshold of heat waves in Estonia is an unusually high air temperature that lasts at least several days. The maximum air temperature was measured at 30°C and over. Therefore, the dangerous air temperature was defined as more than 27°C that lasted several days in Estonia [7].

We also produce the UHI effect data for Tallinn, which can be successfully used for adaptation measures. The intensification of UHI in Tallinn is 5°C according to the study that assessed Landsat-8 images to acquire SUHI. Thus, the threshold of the UHI data is 30-35°C, 35-40°C, and more than 40°C [7].

According to the date of the UHI effect in Tallinn, the related weather data was added to the dataset.

### 2.1 Data Location Description

The location of spatial data is Tallinn, the capital city of Estonia (Fig. 2). The latitude and longitude coordinates are 59.436962 and 24.753574 [1]. The geographical information of Tallinn is 445, 005 population and 159 km^2^ area. The number of city districts in Tallinn is 8 [2]. Moreover, Tallinn is characterized by a humid continental climate with cold winters, according to the Köppen-Geiger classification Dfb [8].

**Fig. 1:**
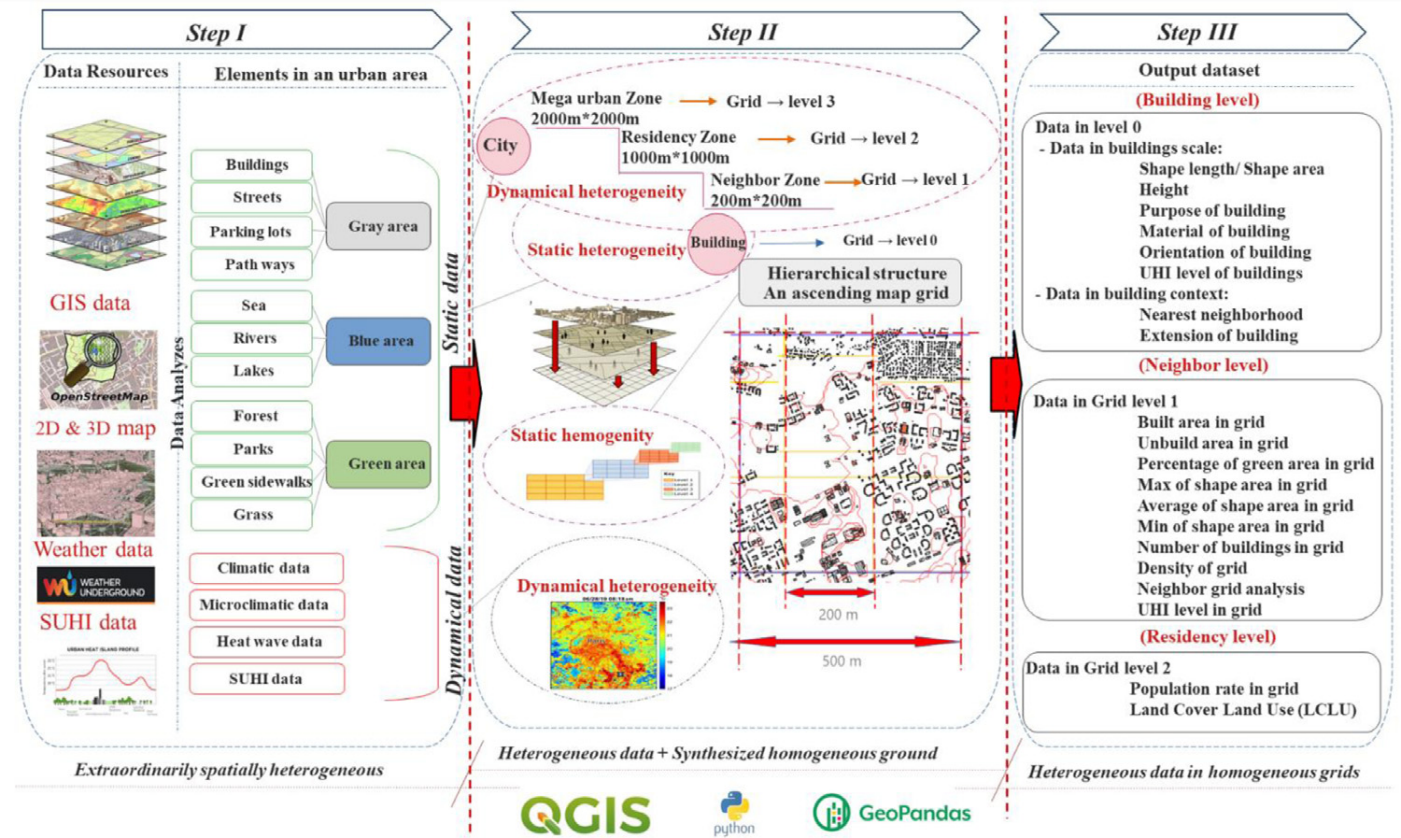
The graphical abstract.

**Fig. 2:**
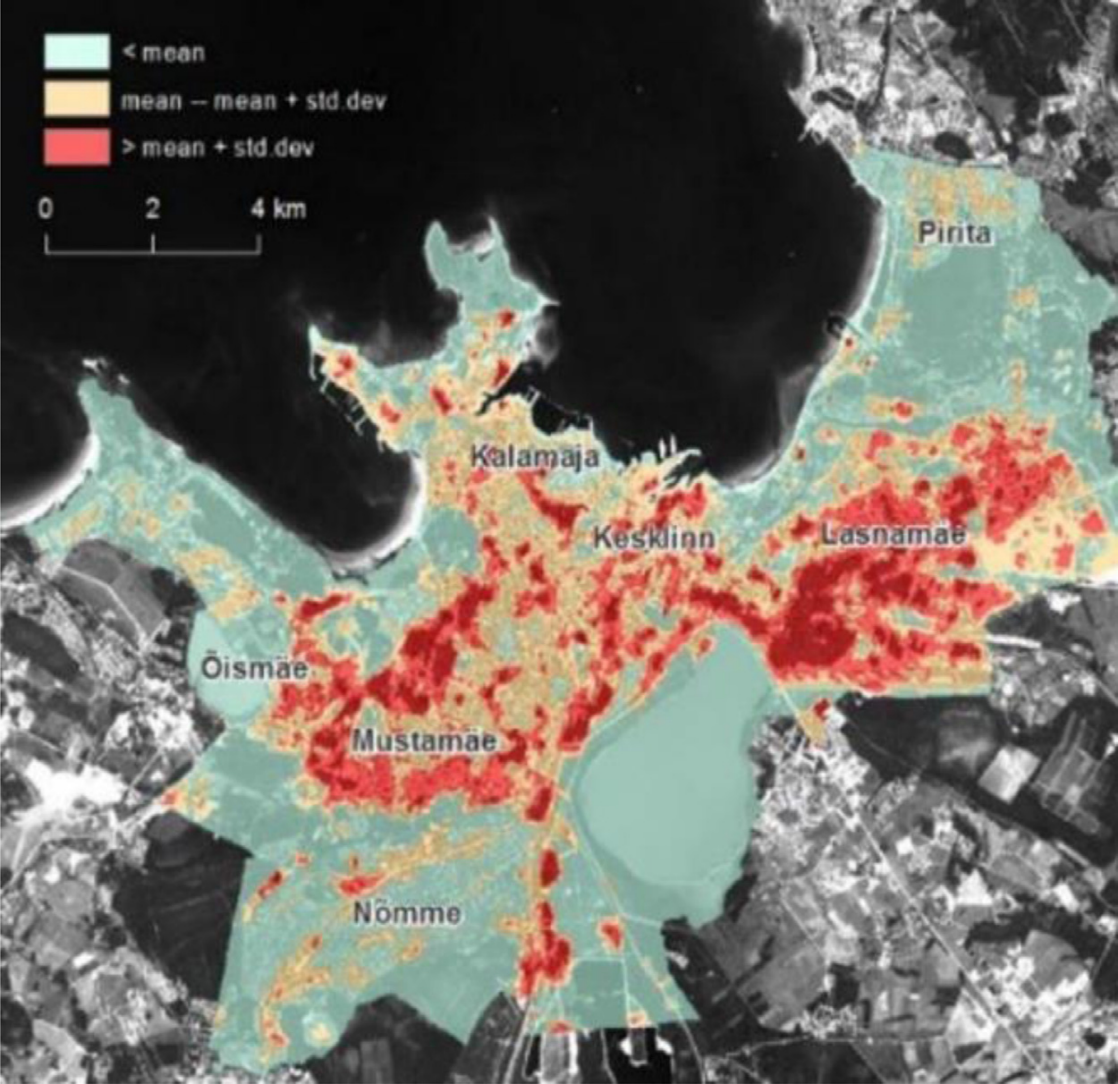
SUHI map of Tallinn, July 25, 2014, 12:30 Estonian summer time (9:30 GMT). The green areas are outside of SUHI, the yellow areas are the areas over the mean SUHI, and the red areas are “inside” the SUHI (over mean + std.dev) [7].

The considered features of buildings are described in Tables 1 and 2. The focused features of buildings in Tallinn are general and technical characteristics. The main features of buildings are the purpose of use, material, absolute height, height, area, length, and a number of floors.

**Table 1:** The description of building data based on Open data information and instructions [9].

Name of features	Description
General data of the building	

Object ID	The ID of each building is a unique combination of numbers
Purpose of use	The purpose of use with the largest area (Table 3)

General technical data of the building	

Material	The main material of the construction: Stone, wood, metal, composite material, and stone, composite material and metal
Absolute height (m)	The highest point of the highest structure
Height (m)	The largest vertical dimension of the building from the ground or pavement immediately surrounding the buildings to the highest point of the highest structure of the building, without taking into account local smaller depressions and elevations
Area(m2)	All building areas are in common use by residential and non-residential users
Length (m)	The length of the shape of the building
Number of floors	The horizontal plane in a building, on which it is possible to use the building according to its purpose

**Table 2:** Purpose of use of buildings.

The purpose of buildings	Description
Residential building	Buildings for dwelling purposes
Public building	Buildings for particular public or common use (such as schools, shopping centers, banks, and offices)
Outbuilding	Buildings that are not used for residential purposes that are not industrial facilities, and not for public use
Underground building	Building with no floors above ground
Industrial facilities	Building for manufacturing and production processes, or a warehouse; a building not for public use
Underground storage space	Storage building with no floors above ground
Parking facilities	Underground garage or parking lot for cars

**Fig. 3:**
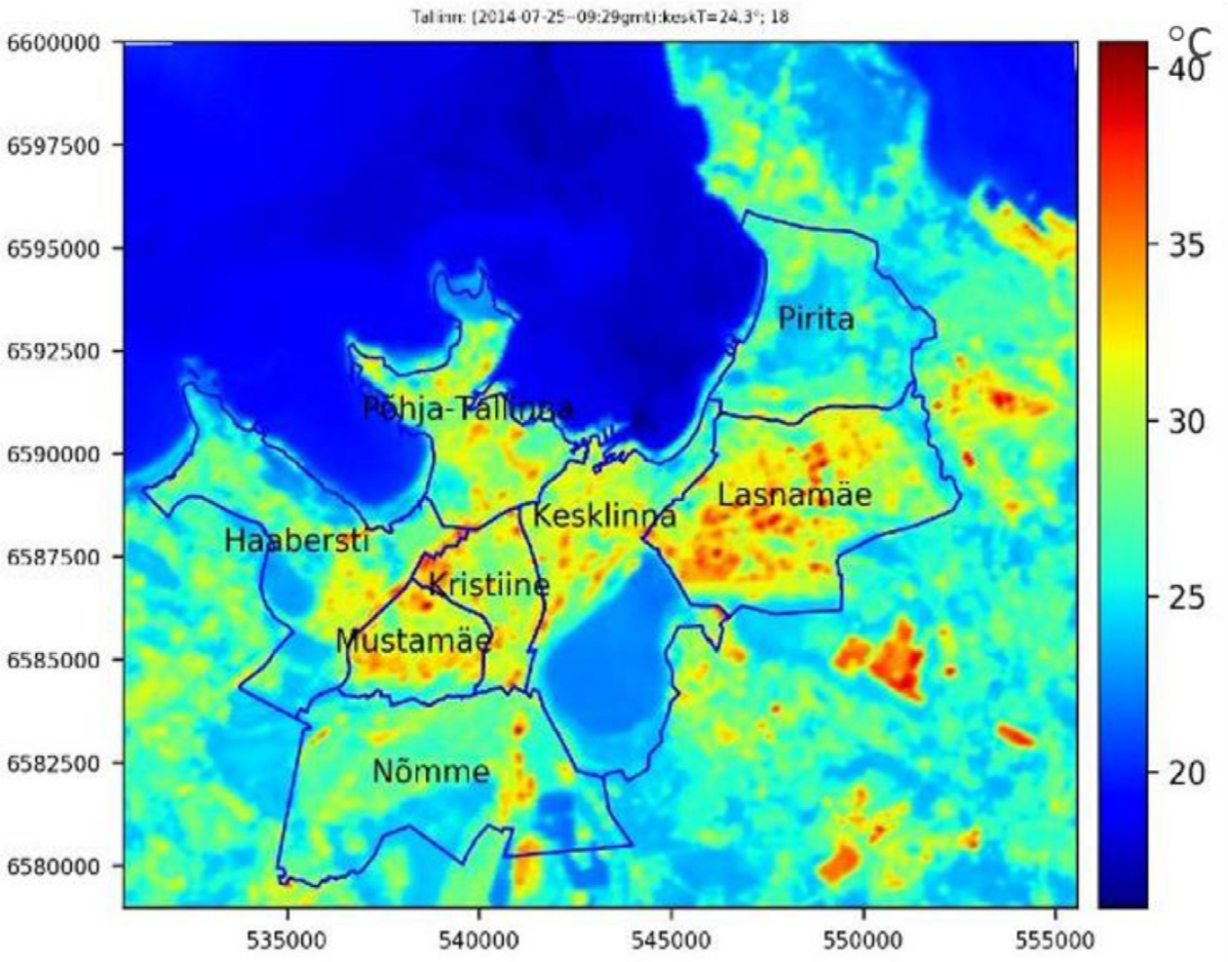
Heat map of UHI, Tallinn July 25, 2014, UHI map of in Tallinn, July 25, 2014, 12:30 Estonian summer time (9:30 GMT) [7].

Furthermore, the weather data is related to the days of UHI value in the summer of 2014-2019. The weather data resource is the underground website [10]. The data of UHI downloaded from the Environment Agency is based on national monitoring data collected by the Environment Agency and analyses carried out on Landsat-8 (USA) satellite data (UHI 2014-2019) [11]. For example, Fig. 3 shows the heat map of the UHI on 25 July 2014. All UHI data used are Shapefiles in the format of (.SHP and .SHX files). The data shows UHI value in Tallinn is categorized into three levels: lower than 30°C (29°C), 30°C and 35°C.

## 3 Experimental Design, Materials and Methods

The framework is tested in an urban assessment practice to collect geospatial data of buildings and their context on the one hand and data related to the UHI phenomenon to perform geoprocessing of the data on the other hand. The geodata is integrated into the latest version of the QGIS Tool (QGIS 3.22.11 ’Białowieża’, 3.26.x Buenos Aires, Ubuntu), and the data analysis is implemented in Python 3 environment in Jupiter notebook interface. To generate the dataset, a three-step methodology is developed:

### 3.1 First Step: Resources of Urban Data

The first step is to consider the available resources of urban data and the schema of all data types. This part of the process starts with defining urban data as the subject of data collection from different complex systems and resources.

The spatial information available in the public domain is downloaded and imported from the Tallinn Geoportal and Geodata [6,9,11].

### 3.2 Second Step: Urban Heterogeneity, Homogeneity, and the Ascending Hierarchical Grid System

The second step refers to the data recognition and categorizing of the data schema into homogeneous or heterogeneous and static or dynamic, then implementing the hierarchical grid system in the environment of the QGIS Tool.

The initial available data combine two components of the schema: static heterogeneity data and dynamic heterogeneity data.

Static heterogeneity means that elements in an urban area, like buildings, streets, and the natural landscape, remain unchanged or change little over time.

Dynamic heterogeneity refers to frequently changing data, such as climate, microclimate, and UHI data.

Here, when data collection does not consider the context of buildings, it is called static heterogeneity. However, when the data is based on urban elements at a larger scale, i.e., considering objects in an urban area in the context, such as characteristics of buildings in their neighborhood, the data is called dynamic heterogeneous data.

The aim of creating the hierarchical grid system is:

First, a spatial index is created for each object on the map, and the objects are referenced to the underlying grids. The grid system aids in analyzing extensive spatial data by effectively dividing the areas of the earth into identifiable grid cells.

Secondly, use the homogeneous ground to define urban indices mainly related to the heterogeneous data, which means accommodating both static and dynamic data in the solid hierarchical grid system.

This means that the hierarchical structure provides a solid basis for collecting data based on their locations. Thus, each grid is characterized by homogeneous static data in one layer and includes all points, geometries, and other recognized elements below the grid. For example, in the definition of built-up area, the total buildings’ area located in grid level 1 is considered (Table 3).

**Table 3:** Defined indices, using a homogeneous hierarchical structure system and heterogeneous urban data.

Index	Grid level	Description
Building data	Grid 200 m*200m level 1	Buildings in 29°C, 30°C and 35°C UHI level
Built up area	Grid 200 m*200m level 1	B = Sum of buildings’ area located in grid level 1
Density	Grid 200 m*200m level 1	D = Sum of all buildings’ area located in grid level 1/40000
Green area	Grid 200 m*200m level 1	G = Sum of the area of green spaces, located in grid level 1

**Fig. 4:**
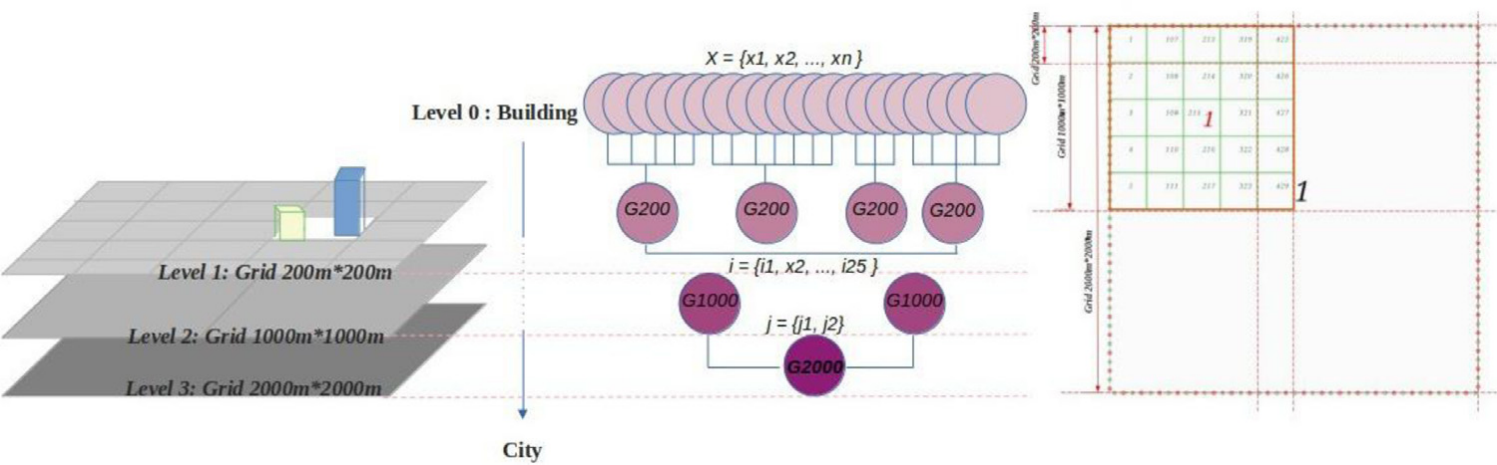
The defined ascending hierarchical grid system.

In this work, the hierarchical grid system in QGIS Tool represents polygons covering all parts of the map of Tallinn in three independent layers. The polygons are defined in three scales of geometric grids in a Shapefile format, as Fig. 4 shows.

As mentioned earlier, the data collection benefits from the hierarchical grid system and analyzes the spatial data of the target area on the map. Each level of the grid system has an ascending hierarchical structure, starting with the smallest scale, the square with a size of 200 meters. The second level of the grid system is a square with a dimension of 1, 000 meters in 1, 000 meters, while the largest level is a square with a dimension of 2, 000 meters in 2, 000 meters. The ascending grids are respectively called from levels 1 to 3:

Neighbourhood Zone, Residential Zone, and Mega Urban Zone, while the base level is the building level (level 0) as the smallest scale (Fig. 4)

The set X= x_1_, x_2_, . . ., x_n_} is the building objects in the building dataset (level 0). First, this set is aggregated to the defined ascending hierarchical grid system. For example, x_i_ is the building located in the i th grid of level 1, so the i th grid is aggregated by the j th grid of level 2 and the k th grid of level 3. Then the spatial index of each building is appended to the dataset to determine the exact position of the building in the ascending hierarchical grid system. For example, Fig. 4 shows that each grid unit of level 3, a grid of 2, 000 meters by 2, 000 meters contain four squares of level 2 grids of 1, 000 meters by 1, 000 meters, and at the same time includes 25 squares of level 1 at 200 meters dimension.

### 3.3 Third Step: Object Detection and Index Definition

The last step of this section is related to the processing, arranging, and collecting data. Here we have used Python libraries and packages on the one hand and benefited from the geoprocessing attributes and expressions of the QGIS Tool, which have helped us develop methods for data collection in the different grid levels, starting from level 0 to level 3.

The hierarchical structure system is used to collect data related to the level of the grid system and defined indices mentioned in Table 3.

### 3.4 Experimental Methods to Capture Data

This subsection summarizes the approach taken to develop the geospatial dataset.

**Data acquisition I: object detection in the hierarchical grid system**

This part aims to show how the UHI value of each building is detected. The next step is completing the dataset by appending more details about buildings. As we mentioned, UHI intensity levels in the main levels of SUHI are:

Lower than 30°C (29 °C);

30 °C;

And 35 °C [7,6].

Moreover, this part's data collection process is done in Grid 2, Level 1 of the hierarchical grid system.

According to the geometric classes of objects in the QGIS Tool, we are dealing with polygons when the objects that should be detected are buildings. Moreover, objects located in the same places can be tracked based on the coordinate reference system (CRS) as a reference system to define the location of features in space [12]. When features are retrieved from a layer, the associated geometries have coordinates in the CRS layer. Therefore, objects on the map on the same CRS have intersections. In the code we developed in Python, we used this relationship to list buildings in each UHI area. The list contains buildings with unique identification codes in the specific ID of UHI. Furthermore, these two layers are in the area covered by the hierarchical grid system.

The codes are developed and executed in Python in the Jupiter Notebook interface. The used libraries are Pandas and Numpy to import the data as a Shapefile and process the imported data. Next, the Geopandas package is imported into the Notebook. The next is object detection, where we used GeoSeries.intersection attributes from the Geopandas package.

Since we need the identification code of each building to append other features in the next step, the method should use the unique code of buildings (ObjectID in the initial data), then find out the intersected geometries and return the ID of the building and the UHI map. Furthermore, in this method, we need to use more than one feature of each object. Therefore, we should consider a function to iterate pairs. For example, the zip () function in Python returns a zip object that is an iterator of tuples where the first element in each passed iterator is paired. Then the second element in each passed iterator is paired.

The for-loop function is used to iterate over a sequence of tuples. The for loop can execute a series of statements, once for each element in the tuple. The "for loop" is repeated twice to build up the dataset and the UHI data with the zipped iterated objects.

The following codes use the intersection attributes of Geopandas, Python's zip function, and find the intersection between two called geometries of the two datasets and the ID of the building that has intersected with the UHI values. Considering the UHI value and the date, we could collect data from buildings with different heat values in the city. Moreover, the codes to determine which building is under which grid are similar to this code.

The codes are given below:

for r, s in zip (Buildingdata.geometry, Buildingdata.ID): for v, o in zip (UHI.geometry, UHI.ID):

Intersection_area = r.buffer(0).intersection(v.buffer(0)) if not intersection_area.is_empty:

p = gpd.GeoSeries(intersection_area) print(f"{int(o)} has intersection with {s}")

**Data acquisition II: the nearest neighbor**

Another important feature in understanding the urban area's density and the city's compactness is the distance between buildings. We aim to find the shortest Euclidean distances between buildings at the neighborhood scale. We can use the spatial index defined in the previous step to determine the size faster than looping through the data frame and then finding the minimum of all distances when working with a large data frame.

As in the previous code, we used the zip () function to return the ID of each building and understand the pair of neighboring buildings and the for-loop function in Python, as well as the distance attributes of the Geopandas package.

According to the codes, the output will be the list of distances, starting with 0 as the minimum distance, i.e., the distance of each building to itself, and the second member of the list is the nearest building. Then we use the list of minimum distances and call the second member (element number of the second item = 1 since the first element is the 0th item in the list).

**Fig. 5:**
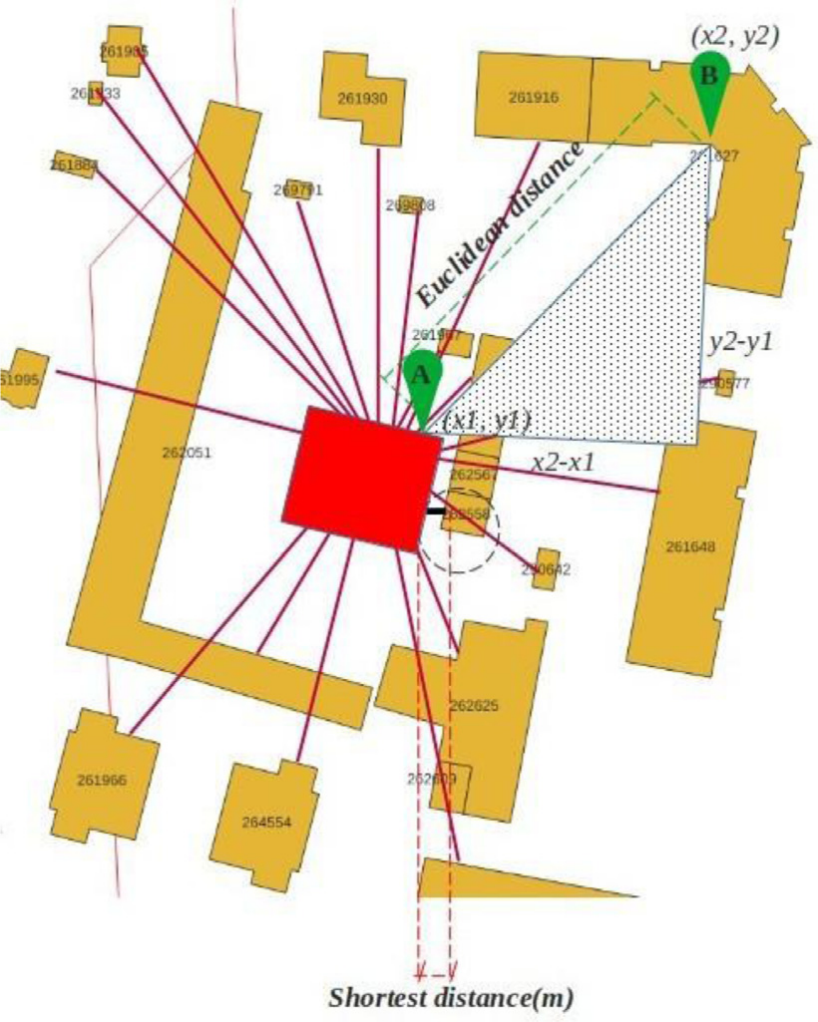
The nearest neighbor to each building.

The codes can also find the distance to other boundaries for any geometry or the furthest neighbor. Furthermore, the method can be useful to find the nearest street, green space, or other location as a geometry, point, or line, even in two different data resources. Fig. 5 shows how the nearest neighbor is defined. First, each building is set as a target geometry to calculate the distance between the outer wall of the building. The distance is the shortest line between two objects. For each x_n_ member of the building dataset, the distance to (n-1) members of the list is calculated. If the distance between the centers of geometries is important, we should calculate the centroids for all the polygons that represent the boundaries of the buildings. The centroids can be calculated using the centroid attributes of the Geopandas package. It can be helpful when the distance between the center of two or more geometries, like cities, is needed.

To calculate distances, we invoke the distance function of the Geopandas package. In the latter, the distance between two objects is usually defined as the smallest Euclidean distance or straight-line distance among the possible distance pairs of the two geometries.

**Fig. 6:**
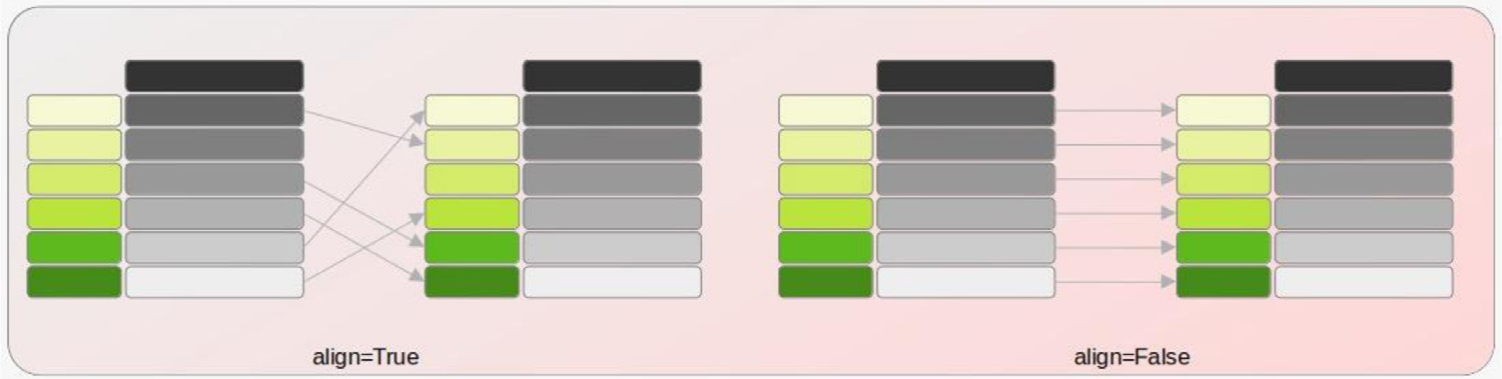
The GeoSeries distance, index value True or False [13].

By applying distance, we can match the index values of the GeoSeries (geometries) and compare elements with the same index with align=True or ignore the index and compare elements based on their matching order with align=False [13] (Fig. 6).

The code back ID of two adjacent buildings with the minimum distance in meters between them. The codes for calculating the nearest neighbor are given below:

for i, y in zip (DFB.geometry, DFB.objectid):

distance = DFB.distance(i, align = True) sorted_list=sorted(distance.to_list()) min_sorted_list=sorted_list[1]

print (f"the nearest neighbour of {int(y)} building ID is located in min_sorted_list} meters")

In addition, if the maximum, mean, and average distance are needed, the following attributes of Geopandas in the codes can be applied.

maximum_distance = distance.max() mean_distance= distance.mean()

**Data acquisition III: main angle and orientation of buildings**

The orientation of the building is of paramount importance factor that affects the incident solar radiation and the absorbed heat wave. According to Mondal, a building with an east-west orientation (EW) has maximum solar gain, and a building with a north-south orientation (NS) has minimum solar gain [14]. Since data collection aims to create a predictable and explainable ML UHI model, the sub-scoring of buildings oriented in NE-SW and NW-SE is crucial because it shows us how buildings are exposed to solar radiation and receives heat wave.

**Fig. 7:**
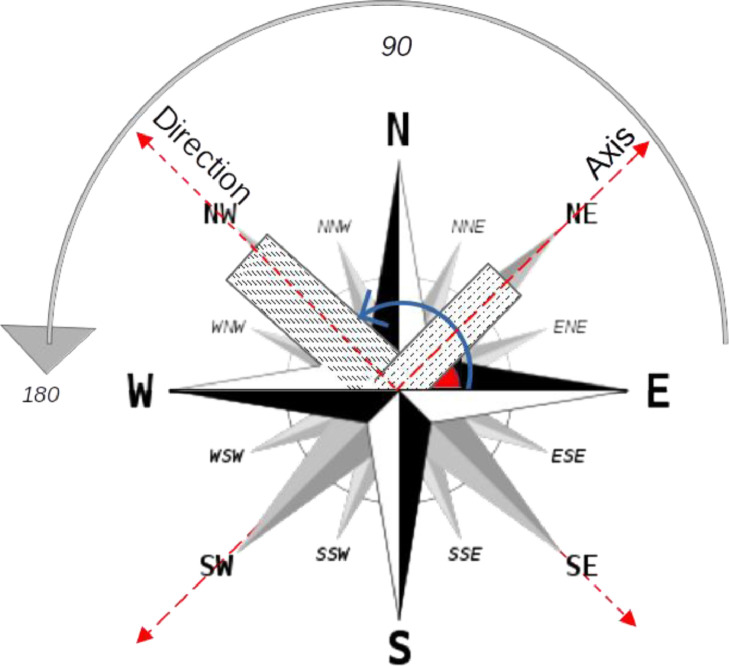
Direction, angle, and orientation of buildings.

To determine the angle values of the building, we need to know the orientation of the longer side of the building and the direction of each building. The conventional axes denote the directions, as Fig. 7 shows. The east direction (positive x-axis) is assumed to be 0 degrees, as shown in Fig. 1. The angles are calculated in an anticlockwise direction, i.e., north is 90 ° and west is 180 ° [14]. The orientation of a building is the direction or angle to which the length is facing. Since the axis of a building is parallel to its length, or conversely, perpendicular to its orientation, the main angle of the building is located on the right side of the following figure is 45°. In contrast, the orientation of the building is 135° in the NE-SW direction. Considering the building on the left side of the figure, the angle is 135°, the orientation is 45°, and the direction is NW-SE.

In addition, Fig. 7 indicates the building direction and angle calculated using QGIS Tools.

The expression is available in the windows of the field calculator.

To determine the main angle of all buildings in the dataset, all geometries should be called with $geometry in the field calculator window of the expression part of QGIS Tools. expression main_angle ($geometry)

### 3.5 Completing the Dataset

The last step to prepare the dataset is appending other general and technical characteristics of buildings in different levels of the hierarchical grid system on a different scale (Tables 1, 2, and 3).

Ultimately, the dataset is created according to the characteristics of the buildings, their spatial indices on the hierarchical system grid, the UHI value of each building, the defined indexes based on buildings and different zones in the city area, and the weather data on dates that the city experienced UHI phenomena.

## 4 Study Limitations

This work is subject to the following limitations in exploring and acquiring urban data, especially when creating a comprehensive dataset that covers various essential features related to buildings, their surroundings, and other environmental factors in different scales.

Some of the limitations that we faced while collecting urban data are:

Lack of data sources: In data acquisition on the urban scale, there is a need for more data sources for specific features related to buildings and their surroundings.

Need for more data: Even if data sources are available, in some cases, the information may need to be completed or detailed enough to create a comprehensive dataset. For example, the available data on green areas or open spaces in the city need to be more detailed to capture all the necessary information about the vegetation cover or the quality of the environment. Data quality: Another limitation of urban data acquisition is its quality. Generally, data collecting needs to be more accurate and complete, which can affect the accuracy and reliability of the dataset. For example, there is just some general information, not detailed data, about some of the features of buildings, like the shape of the roofs, compositions of facade materials, and morphology of buildings.

In addition, there are other challenges that one might face when collecting and analyzing urban data. For example, collecting data on some features, such as the shape of the roof of a building or the features of neighbors, require on-site inspections, which are time-consuming and costly. Moreover, some data sources, such as GIS and geo data, must provide the level of detail required to capture the necessary information accurately.

## Ethics Statement

The authors declare that the hereby presented data and data article fully comply with the Journal's policy regarding authors’ duties, data integrity, and experimental requirements.

## Credit Author Statement

**Nasim Eslamirad:** Conceptualization, Methodology, Programming, Data capturing, Writing – Original draft preparation; **Francesco De Luca:** Conceptualization, Supervision, Reviewing, and Editing; **Kimmo Sakari Lylykangas:** Supervision; **Sadok Ben Yahia:** Conceptualization, Supervision, Programming, Reviewing, and Editing; **Mahdi Rasoulinezhad:** Conceptualization, Programming.

## Declaration of Competing Interest

The authors declare that they have no known competing financial interests or personal relationships that could have appeared to influence the work reported in this paper.

## Data Availability

Geoprocess of geospatial urban data in Tallinn, Estonia (Original data) (Mendeley Data). Geoprocess of geospatial urban data in Tallinn, Estonia (Images) (Original data) (Mendeley Data). Geoprocess of geospatial urban data in Tallinn, Estonia (Images) (Original data) (Github).
